# PSMA-targeted therapy for non-prostate cancers

**DOI:** 10.3389/fonc.2023.1220586

**Published:** 2023-08-14

**Authors:** Jarey H. Wang, Ana P. Kiess

**Affiliations:** Department of Radiation Oncology and Molecular Radiation Sciences, School of Medicine, Johns Hopkins University, Baltimore, MD, United States

**Keywords:** PSMA, radioligand therapy (RLT), non-prostate cancers, lutetium-177, salivary gland cancer, glioblastoma, renal cell carcinoma, soft tissue sarcoma

## Abstract

Radioligand therapy (RLT) agents are demonstrating a crucial role in the clinical approach to aggressive malignancies such as metastatic castrate-resistant prostate cancer (m-CRPC). With the recent FDA approval of prostate-specific membrane antigen (PSMA)-targeted RLT for m-CRPC, the field has broadened its gaze to explore other cancers that express PSMA in the tumor parenchyma or tumor neovasculature. In this review article, we discuss current progress in the clinical use of PSMA RLTs in non-prostate cancers such salivary gland cancers, renal cell carcinoma, high grade glioma, and soft tissue sarcoma. We highlight early reports in small case series and clinical trials indicating promise for PSMA-targeted RLT and highlighting the importance of identifying patient cohorts who may most benefit from these interventions. Further study is indicated in non-prostate cancers investigating PSMA RLT dosimetry, PSMA PET/CT imaging as a biomarker, and assessing PSMA RLT safety and efficacy in these cancers.

## Introduction

In the era of personalized medicine, theranostic approaches are continuing to evolve and shape the clinical landscape for many cancer types. In particular, radioligand therapies (RLT) comprised of radioactive isotopes conjugated to peptide ligands are allowing for targeted approaches for aggressive neoplasms, such as metastatic castrate-resistant prostate cancer (m-CRPC). These hybrid therapies offer a unique modality to deliver localized radiation to endogenous targets, and are not only safe but also efficacious (1, 2). The recent phase III VISION trial of patients with m-CRPC who failed hormone and taxane therapies demonstrated a significant increase in median progression-free survival (8.7 vs. 3.4 months) and overall survival (15.3 vs. 11.3 months) when ^177^Lu-PSMA-617 (lutetium-177 conjugated to prostate-specific membrane antigen-617 targeting ligand) was added to standard of care^2^. The efficacy of ^177^Lu-PSMA-617 has generated interest in applying PSMA RLTs earlier in prostate cancer management, as well as in other cancer types in which PSMA is expressed.

In this review article, we discuss current progress in the clinical use of PSMA RLTs in non-prostate cancers. We highlight early reports in small case series and relevant ongoing early clinical trials, in particular recent progress in salivary gland cancers, renal cell carcinoma, high grade glioma, and soft tissue sarcoma. We also discuss the limitations and future directions for this growing field.

### PSMA expression in non-prostate malignancies

Prostate-specific membrane antigen (PSMA), a type II transmembrane glycoprotein, was first described as highly expressed in benign and malignant prostate epithelium (3, 4), with substantially higher PSMA expression in prostate adenocarcinoma compared to normal tissue (5, 6). However, contrary to its name, PSMA also exhibits expression in several non-prostatic tissues including salivary glands, kidneys, and gastrointestinal mucosa (7, 8). Expression of PSMA has also been reported in the tissues of many solid tumors and their neovascular endothelium (9). PSMA has a functional role in promoting angiogenesis within tumors, a process mediated by laminin substrates to promote endothelial cell activation (10). In contrast, this function is absent in normal tissue endothelium. In most solid tumors, the percentages of tumor neovascular endothelium with positive PSMA typically exceed that of the tumor cells themselves, as is the case for primary brain tumors, lung cancer, breast cancer, gastrointestinal tumors, and renal cell carcinoma (RCC) (9, 11).

Certain tumors like pancreatic ductal adenocarcinoma (PDAC) and salivary gland tumors also express PSMA in a significant proportion of the tumor parenchyma. PDACs express PSMA in 67% of cases, with higher expression possibly associated with worse survival outcomes (9, 12). However, clinical applications of PSMA RLTs in PDAC have yet to be investigated. In contrast, PSMA RLTs for salivary gland cancers (adenoid cystic carcinoma and salivary ductal carcinoma) have garnered significant interest. PSMA levels are detectable in the majority (as high as 90%) of certain salivary gland tumors by PSMA PET/CT, which has been demonstrated in small cohort clinical trials (13, 14).

### Types of radionuclide emitters

Currently, the small molecules PSMA-I&T and PSMA-617 are the most developed PSMA-targeted small molecule therapies. Both are conjugated to lutetium-177 (^177^Lu). While the two ligands are fairly dissimilar apart from the urea-binding motif, it appears that in m-CRPC, the toxicity and efficacy profiles are generally similar (15). Both conjugates likely kill tumor cells with the same mechanism. After localization to the tumor, they rely on the beta-emitter properties of ^177^Lu to induce DNA damage. Radioisotopes emitting beta particles (β-), high speed electrons ejected from nuclei during radioactive decay, have certain physical, biological, and dosimetric properties that are particularly suitable for RLTs (16, 17). ^177^Lu emits photons detectable on imaging (SPECT or planar) and has a desirable half-life of 6.6 days, enabling practical shelf life and biologic effect. Beta particles can penetrate tissue in the millimeter range, in contrast to alpha (40-100 µm range) and Auger (<1 µm range) emitters. For non-prostate tumors with heterogeneous PSMA expression or in which the majority of PSMA expression occurs in the neovasculature, this may be preferable in order to maximize impact on tumor tissue and bystander-mediated cell killing (18). However, the higher linear energy transfer of alpha emitters may have benefit for tumor types with lower but more homogeneous PSMA expression. The relative strengths and limitations of different types of emitters for different applications is an area of ongoing discussion and investigation in the field.

### PSMA imaging

The use of PSMA RLTs is typically guided by the ability to detect PSMA-positive tumors via PET/CT using gallium-68 PSMA radioligands (^68^Ga-PSMA) or piflufolastat F 18 (^18^F-DCFPyL). Numerous studies have established PSMA PET/CT as a potential screening modality for patient selection in early stage RLT trials in these non-prostatic cancers (9, 19). Overall, the SUV_max_ values for non-prostate malignancies appear lower than for prostate cancer. However, data on the sensitivity of specific SUV cutoffs are generally lacking. As a result, current trials often require detectable disease on PSMA PET/CT with tumor/liver SUV ratio greater than 1.

## Materials and methods

The materials for this review were obtained through a systematic search of existing literature. Studies describing therapeutic administration of PSMA-based therapy were queried using the following terms in PubMed: (Lutetium-PSMA OR Lu-PSMA OR Lu-EB-PSMA OR Lu-prostate) AND (therapy OR radioligand OR radiopharmaceutical OR theranostic), and existing reviews of RLT in non-prostate cancers were cross-referenced (20, 21). Studies describing RLT use (**Table 1**) were found for salivary gland tumors (14, 22–26), high grade glioma (27–30, 37), sarcoma (31, 32), thyroid carcinoma (33, 34), breast cancer (35), and hepatocellular carcinoma (36). Ongoing clinical trials (**Table 2**) were collated by searching ClinicalTrials.gov for “PSMA 177Lu”. Search results were current as of June 30^th^, 2023. The PRISMA flow chart outlining the search process is depicted in **Supplemental Figure 1**.

**Table 1:** Reports on patients treated with PSMA radioligand therapy (RLT) for non-prostate malignancies.

Cancer type	Author (year), location	Study type	Number patients treated	Therapeutic agent	Radioligand activity and cycles	Dosimetry	Grade 3+ adverse events	Outcomes
Adenoid cystic carcinoma	Simsek et al. (2019) (22), Turkey	Case report	1	^177^Lu-PSMA	7.5 GBq, 1 cycle	High tumor uptake on scintigraphy	None	Pain reduction
Salivary gland cancer	Klein Nulent et al. (2021) (23), Netherlands	Retrospective cohort	4 (ACC) 1 (adenocarcinoma NOS) 1 (acinic cell carcinoma)	^177^Lu-PSMA-617	6.0-7.4 GBq, 4 cycles (n=2), 2 cycles (n=3), 1 cycle (n=1)	NR	Grade 3 thrombocytopenia (n=1)	Symptom relief (n=4); stable disease for 6 and 10 mo (n=2)
Adenoid cystic carcinoma	Wang et al. (2022) (14), China	Prospective trial	4	^177^Lu-EB-PSMA-617	1.85 GBq, 3 cycles (n=1), 1 cycle (n=3)	NR	None	Pain relief (n=4); CR for meningeal met (n=1, 3 cycles); variable reduction in lesion SUV_max_ (n=3)
Salivary gland cancer	Civan et al. (2023) (24), Germany	Retrospective cohort	4 (ACC) 1 (acinic cell carcinoma)	^177^Lu-PSMA-617	5.8-7.6 GBq, 6 cycles (n=1, acinic cell), 2 cycles (n=1), 1 cycle (n=3)	Dose to kidneys and lesions reported	None	RLT halted for 3 patients due to poor dosimetric uptake; PFS 3 mo (n=1); PFS not reached (n=1, acinic cell)
Salivary duct cancer	Terroir et al. (2023) (25), France	Case report	1	^177^Lu-PSMA-617	6.1 GBq, 4 cycles with 2 more planned	Dose to lesion and major organs reported	Grade 3 lymphopenia	Pain reduction and decreased asthenia after cycle 1; stable disease
Salivary gland cancer	Van Herpen et al. (2023) (26), Netherlands	Prospective trial	10 (ACC) 2 (SDC)	^177^Lu-PSMA-I&T	7.4 Gbq, 1 cycle (n=2), 2 cycles (n=4), 4 cycles (n=6)	Assessed but not reported in interim analysis	Grade 3 lymphocytopenia (n=1) and hyponatremia (n=1)	Median PFS in ACC 6.7 mo (OS not reached); early PD in SDC patients
Glioblastoma	Kunikowska et al. (2020) (27), Poland	Case report	1	^177^Lu-PSMA-617	8.39 Gbq, 1 cycle	Dose to lesion and major organs reported	NR	NR
Glioblastoma	Kumar et al. (2020) (28), India	Case report	1	^177^Lu-PSMA-617	3.7 Gbq, 3 cycles	NR	NR	Improved ECOG PS (4 to 3) and decreased lesion size
High grade glioma	Graef, Truckenmueller & colleagues (2022) (29), Germany	Retrospective cohort	3	^177^Lu-PSMA	5.74-6.10 GBq, 2 cycles	Dose to lesion and major organs reported	None	Progression at 6 weeks (n=1); follow up data not matured
Glioblastoma	More et al. (2023) (30), Germany	Case report	1	^177^Lu-PSMA	3 Gbq and 1.8 Gbq (2 cycles total)	High tumor uptake on scintigraphy	None	Partial response at 3 mo; 8 mo OS
Leiomyosarcoma	Jüptner et al. (2019) (31), Germany	Case report	1	^177^Lu-PSMA-617	6.0 GBq, 1 cycle	Modest uptake in gluteal lesion; weak uptake in metastases	None	RLT halted after cycle 1 due to poor uptake
Leiomyosarcoma (uterine)	Digklia et al. (2022) (32), Switzerland	Case report	1	^177^Lu-PSMA-I&T (given with nivolumab)	NR, 2 cycles	Strong uptake in lung metastases	None	Mixed response; index lesion partial response with progression elsewhere
Differentiated thyroid cancer	Assadi (2019) (33), Iran	Case report	1	^177^Lu-PSMA	7.4 Gbq, 1 cycle	Good uptake post therapy	None	Improved respiratory function; died of MI 2 weeks later
Differentiated thyroid cancer	de Vries et al. (2020) (34), Netherlands	Retrospective cohort	2	^177^Lu-PSMA-617	6.0 GBq, 2 cycles	NR	NR	Partial response at 5 wks with progression at 7 mo (n=1); progression (n=1)
Triple negative breast cancer	Tolkach et al. (2018) (35), Germany	Case report	1	^177^Lu-PSMA	7.5 GBq, 2 cycles	High tumor uptake on scintigraphy	None	Progression 1 mo after cycle 2
Hepatocellular carcinoma	Hirmas et al. (2020) (36), Germany	Case report	1	^177^Lu-PSMA-617	5.9 GBq, 1 cycle	Poor tumor uptake on scintigraphy	None	RLT halted after cycle 1 due to poor uptake

NR, not reported; ACC, adenoid cystic carcinoma; SDC, salivary duct cancer; NOS, not otherwise specified; SUV, standardized uptake value; CR, complete response; PD, progressive disease; PFS, progression free survival; ECOG PS, European Cooperative Group Performance Score; MI, myocardial infarction.

**Table 2:** PSMA radioligand therapy clinical trials for non-prostate malignancies.

Cancer type	Trial	Trial posted	Location	Primary aim	Estimated enrollment	Therapeutic agent	Dose
ACC	Hopkins/Stanford, Phase II single arm, multicenter	To be posted	USA	Therapeutic	30	^177^Lu-PSMA-I&T	6.8 GBq every 8 weeks up to 4 cycles; dosimetry cohort first
ACC, SDC	NCT04291300, Phase II single arm, single center	3/2/20	Netherlands	Therapeutic	15	^177^Lu-PSMA-I&T	7.4 GBq every 6 weeks up to 4 cycles
ACC	NCT04801264, single arm, single center	3/16/21	China	Diagnostic (^68^Ga-PSMA)	40 (for imaging arm)	^177^Lu-EB-PSMA-617	1.85 GBq every 8 weeks up to 3 cycles
RCC	NCT05170555, single arm, single center	12/28/21	China	Diagnostic (^68^Ga-PSMA)	40 (for imaging arm)	^177^Lu-EB-PSMA-617	1.85 GBq every 8 weeks up to 3 cycles
HGG	NCT05644080, Phase II single arm, multicenter	12/9/22	Norway, Taiwan	Therapeutic	10	^177^Lu-PSMA-I&T	Not listed; every 8 weeks 3 cycles initially, possible extension to 6 cycles
STS	NCT05420727, Phase 1, single arm, single center	6/15/22	Switzerland	Therapeutic	20	^177^Lu-ITG-PSMA-1*	Not listed; every 6 weeks for 2 cycles
Multiple (basket trial)	NCT05867615 (LUBASKET), Phase II single arm, multicenter	5/22/23	Italy	Therapeutic	100	^177^Lu-PSMA-I&T	7.4 GBq (no risk factors for toxicity) or 5.5 GBq (1+ risk factor for toxicity) every 8 weeks up to 4 cycles

ACC, adenoid cystic carcinoma; SDC, salivary duct carcinoma; RCC, renal cell carcinoma; HGG, high grade glioma; STS, soft tissue sarcoma.

*generic formulation of ^177^Lu-PSMA-I&T.

### Salivary gland malignancies

Investigation of PSMA RLTs for non-prostate cancers has continued to grow in recent years, with early phase clinical trials ongoing for salivary gland malignancies (**Table 2**). Salivary gland carcinomas (SGCs) represent up to 6% of all head and neck malignancies (38), and are categorized into more than 20 histologic subtypes. The majority of RLT development to date has focused on adenoid cystic carcinoma (ACC) and to a lesser extent salivary duct carcinoma (SDC).

ACC arises from the major and minor salivary glands, with a classic cribriform histology, though tubular and solid components can also be present (39). The 10-year overall survival (OS) for ACC has been reported at 37-65% (40). The standard treatment for resectable disease consists of surgery, often followed by radiation to improve local control. However, ACC has a predilection for perineural invasion and recurrence. Distant metastatic progression (commonly to lung, bone, and liver) is the leading cause of mortality, and occurs in nearly half of patients (40). The majority of ACC patients who develop distant metastases do so within the first 5 years (41), after which the median OS is 20-32 months (42). Unfortunately, response rates to chemotherapy and targeted inhibitors (e.g. anti-VEGF) are poor in the progressive/metastatic setting (38, 43). Hence, alternative targeted approaches including PSMA RLTs are being pursued. More than 90% of recurrent or metastatic ACC tumors express PSMA detectable on PET/CT (SUV_max_ range of 1.1-30.2) (13, 44). Additionally, histology shows >50% of cells in primary tumors and up to 92% of cells in metastatic lesions express PSMA (44).

Salivary ductal carcinoma (SDC) is rarer than ACC and typically diagnosed at a more advanced stage. Histologically, SDC resembles ductal carcinoma of the breast, with the majority expressing androgen receptor (AR) and/or Her-2 receptors (9). SDC is an aggressive malignancy, with 3-year OS of around 36% (45, 46). For local disease, treatment typically consists of surgical resection followed by adjuvant therapy with radiation or chemoradiation. For recurrent or metastatic disease, recent retrospective and early stage clinical studies support a role for targeted therapies (47), including androgen deprivation and trastuzumab (46). Recurrent or metastatic SDC also express PSMA detectable on PET/CT, which was reported in a prospective phase II study for 10 patients (SUV_max_ range of 0.3-25.9) (13), with 40% of patients showing a tumor/liver ratio >1 in all tumor sites.

Data on the usage of PSMA RLTs in salivary gland tumors come from case reports and small cohort studies (**Table 1**) (14, 22–26). An early report from Simsek et al. described a patient with adenoid cystic carcinoma that received ^177^Lu-PSMA (7.5 GBq) for palliation of painful bone metastases (22). The patient reported pain relief without notable side effects after one cycle but ended up dying 6 weeks later. Subsequently, Klein Nulent et al. reported retrospective data from patients treated with up to 4 cycles of ^177^Lu-PSMA-617 (6.0-7.4 GBq) on a compassionate use protocol (23). Among the patients treated four had ACC, one had salivary gland adenocarcinoma (not otherwise specified), and one had acinic cell carcinoma. Two patients – both with ACC – completed 4 cycles of therapy. One showed stable lung metastases and minimal progression of liver disease. The other had stable disease with decreased SUV_max_ (from 6.5 to 4.5) and was alive 10 months after treatment start, at which point progression was noted on PSMA PET/CT. Of the 4 patients that progressed on therapy, 2 stopped after cycle 2, one stopped after cycle 2 due to grade 3 thrombocytopenia (the only grade 3 event), and one stopped due to demotivation from side effects after cycle 1. Notably, 4 patients reported reduction in tumor-related symptoms, particularly pain, after the first cycle.

More recent studies have also begun to report dosimetry details. Terroir et al. described treatment of one patient with SDC with ^177^Lu-PSMA-617 on a compassionate use protocol (25). The patient received 4 cycles for a cumulative dose of 24.3 GBq, which was well tolerated with grade 3 lymphopenia being the worst adverse effect. The patient reported improvement in bone pain after 1 cycle. The index femoral head lesion (PSMA PET/CT SUV_max_ of 10 pre-treatment) received 0.04 Gy/GBq, and was stable on follow up PSMA PET/CT. A separate study by Civan et al. also reported 5 patients (4 with ACC and 1 with acinic cell carcinoma) treated with ^177^Lu-PSMA-617 (5.8-7.6 GBq) (24). The absorbed dose to index lesions ranged from 0.06-0.68 Gy/GBq on SPECT/CT. Due to poor lesion PSMA uptake on 24-hour post-treatment scintigraphy, RLT was discontinued for 3 patients with ACC. One patient with ACC had a progression free survival of 3 months after 2 cycles. The patient with acinic cell carcinoma had continued response after 6 cycles, with accumulated dose of 0.41 and 0.49 Gy/GBq for two index lesions after cycle 1.

There are two early-stage clinical trials for recurrent/metastatic ACC that are enrolling and one that will begin enrolling soon (**Table 2**). The forthcoming Hopkins/Stanford trial and NCT04291300 (Dutch) are phase II single arm studies and NCT04801264 is an early phase study. The primary endpoint of the NCT04801264 study is diagnostic evaluation of ^68^Ga-PSMA-617. The trial also incorporates a pilot study of ^177^Lu-EB-PSMA-617 therapy (1.85 GBq), a derivative of ^177^Lu-PSMA-617 in which the Evans blue (EB) motif helps to bind albumin and slow plasma clearance, which may promote tumor accumulation (14). This therapy was ultimately received by 4 patients, with one patient receiving 3 cycles and three patients receiving only 1 cycle (due to restrictions during the COVID pandemic). Subjective pain symptoms were improved for all 4 patients and no grade 3 side effects were reported. The patient who received 3 cycles had a significant reduction in SUV_max_ (from 7.0 to 1.1) in a solitary meningeal metastasis. Metabolic responses after cycle 1 were variable, however. One notable finding was that diagnostically, ^68^Ga-PSMA-617 outperformed traditional ^18^F-FDG PET except in lung metastases, which may have implications for patient selection given that ACC most commonly metastasizes to lung.

The phase II Dutch trial is a single-center study using ^177^Lu-PSMA-I&T dosed at 7.4 GBq every 6 weeks up to 4 cycles and includes both ACC and salivary ductal carcinoma. The Hopkins/Stanford trial will be a multi-center study using ^177^Lu-PSMA-I&T dosed at 6.8 GBq every 8 weeks up to 4 cycles for ACC. Enrollment in both require detectable disease on PSMA PET/CT (with tumor/liver ratio greater than 1). The Dutch trial recently reported results for the first 12 patients treated (10 ACC and 2 SDC) (26). RLT was discontinued for two patients after 1 cycle and 4 patients after 2 cycles due to early progression. Six patients with ACC received 4 cycles, with median PFS of 6.7 months (3 patients with stable disease and 3 with progression on 3-month imaging). The treatment was well tolerated, with two grade 3 toxicities: lymphopenia and hyponatremia. Dosimetry results were not yet reported.

### Renal cell carcinoma

The clinical role of PSMA RLT in renal cell carcinoma (RCC) is being investigated in an early phase trial NCT05170555 (**Table 2**). RCC originates from the renal epithelium and accounts for >90% of kidney cancers. Within RCC, most have clear cell histology. RCC is relatively resistant to conventional chemotherapy and radiation, with current median OS of 13 months for metastatic disease. While targeted therapies against mTOR and VEGFR and immunotherapies are approved in metastatic RCC, they have limited efficacy (48). RCC is a highly vascular tumor, and PSMA is over-expressed in the neovasculature in the majority of tumors. Expression occurs primarily in clear cell (84% on average) and chromophobe subtypes (61% on average), whereas papillary and transitional RCC have <30% endothelial expression by IHC (9, 11, 49, 50). Increased PSMA expression may be associated with adverse pathologic features and worse overall survival (51, 52). ^68^Ga-PSMA PET/CT has been shown to have greater sensitivity than conventional modalities for detecting metastatic disease, particularly for clear cell (ccRCC) (50, 53). For metastatic ccRCC lesions, SUV_max_ values have been reported in the range of 1.2-48, with lung metastases appearing to have lower SUV_max_ values than other sites (54–56).

Given these promising data, several early stage trials are in progress to determine the role of PSMA PET/CT in diagnosis and restaging of advanced RCC (57). To date, no case reports for RCC patients treated with PSMA RLTs exist. The NCT05170555 trial has a primary goal of assessing the diagnostic value of ^68^Ga-PSMA PET/CT. Additionally, it will evaluate the safety and efficacy of ^177^Lu-EB-PSMA-617, which will be dosed at 1.85 GBq every 8 weeks up to 3 cycles in select patients based on PSMA avidity. Renal dosimetry and toxicity may be important for these studies.

### High grade glioma

The role of PSMA RLTs in the setting of recurrent high-grade glioma is under evaluation in the phase II trial NCT05644080 (**Table 2**). PSMA is highly expressed in the neovasculature of high-grade gliomas, due to the increased vascularity compared to low-grade glioma. For glioblastoma multiforme (GBM), tumor vascular epithelia demonstrate PSMA expression in all cases compared to only 5% of tumor cells (9). With standard treatment consisting of surgery followed by chemoradiation, the median OS for GBM is 15 months. After recurrence or progression, effective options are limited. Six patients to date have been treated with ^177^Lu-PSMA-617 in the setting of refusing or exhausting standard therapeutic options (**Table 1**) (27–30, 37). The first case reported by Kunikowska et al. was for recurrent GBM previously treated with surgery and chemoradiation, with SUV_max_ of 10.3 on ^68^Ga-PSMA-11 PET/CT (27). One dose (8.39 GBq) was given with estimated 14 Gy delivered to the tumor and limited dose to other organs. No outcomes were reported. Kumar et al. described a second patient with recurrent GBM who received 3 cycles of 3.7 GBq at 2 month intervals (28). No adverse effects were reported, and the patient had improvement in performance score and reduction in lesion size. Recently, More et al. reported a case of recurrent GBM treated with 2 cycles of ^177^Lu-PSMA, 3.0 GBq followed by 1.8 GBq (30). The tumor PSMA PET/CT SUV_max_ was 10.2 (compared to 2.9 for liver) and post-treatment scintigraphy showed good uptake. The treatment was well tolerated. The tumor showed partial response at 3 months, and the patient survived for 8 months after initiation of RLT.

The aforementioned cases suggest good CNS penetrance for PSMA RLT, though recent dosimetry studies from a retrospective cohort by Graef, Truckenmueller and colleagues raise concerns that delivered dose and efficacy may be lower than previously described (29, 37). The authors screened for eligible patients with high grade glioma using ^68^Ga-PSMA-11 and found that for a tumor-to-background (liver) cutoff ratio > 1, only 3 of 20 patients (15%) met criteria. The tumor SUV_max_ values for these patients were 8.65, 7.97, and 6.39. Patients were treated with ^177^Lu-PSMA (5.74-6.10 GBq) up to 2 cycles with median dose of 0.56 Gy. At time of reporting, only one patient underwent follow up imaging at 6 weeks, which showed tumor growth. Overall, the treatments were well tolerated.

### Soft-tissue sarcoma

The phase I trial NCT05420727 is currently aimed at testing the feasibility of PSMA RLT in soft tissue sarcomas (**Table 2**). Soft tissue sarcomas arise from connective tissues, such as muscle, fat, nerves, lymphatics, and blood vessels. Due to the heterogeneity of presentation, sarcoma management varies depending on histology, though typically consists of gross total resection, when possible, along with chemotherapy and/or radiation. Recurrences and metastatic disease are notoriously difficult to treat. PSMA expression in soft tissue sarcoma occurs primarily in the neovasculature and varies based on subtype. From IHC studies, reported values range from 23% of dedifferentiated liposarcoma to >50% of pleomorphic rhabdomyosarcoma and liposarcoma (58). Uptake of ^68^Ga-PSMA-11 on PET/CT also varies based on histology (59), with reports of liposarcomas showing PSMA uptake up to SUV_max_ of 13 (60) and metastatic leiomyosarcoma SUV_max_ of 16.5 (31). There have also been two case reports of sarcoma patients treated with PSMA RLT. In one case, a patient with progressive metastatic leiomyosarcoma was considered for ^177^Lu-PSMA-617 as part of a compassionate use protocol after refusing chemotherapy. She was treated with one dose of 6.0 Gbq, which was reportedly well tolerated (31). However, whole body scan revealed only weak uptake of the radiotracer, and hence the therapy was discontinued. Follow up PSMA PET/CT three months later showed disease progression. For the second case, a patient with metastatic uterine leiomyosarcoma was treated with a combination of ^177^Lu-PSMA-I&T in 8 week intervals and nivolumab given 1 week after each RLT treatment for 2 cycles (32). PSMA PET/CT 4 months after treatment initiation showed reduction of primary tumor growth rate of initially reported lesions, with reduction in size of the lung nodule with highest uptake (SUV_max_ 8.9). However, due to progression of a neighboring lesion with low uptake, the patient was switched to next line chemotherapy.

### Other non-prostate malignancies

While not being studied in clinical trials yet, the use of PSMA RLTs have also been reported in thyroid cancer, hepatocellular carcinoma, and breast cancer (21).

Thyroid malignancies also express PSMA in the neovasculature (but not tumor cells) of all histologic subtypes (21). Three patients have been treated with PSMA RLTs to date (33, 34). One patient had differentiated thyroid cancer (DTC) that progressed after multiple lines of therapy, including radioactive iodine, sorafenib, and ^177^Lu-DOTATATE ^(^ 33). The patient had severe dyspnea due to progression in the neck and lungs and was reported to have some improvement in respiratory function following 7.4 GBq of ^177^Lu-PSMA. However, he died unexpectedly from cardiac arrest 2 weeks following treatment. In a separate study, two patients underwent ^177^Lu-PSMA-617 for treatment-refractory DTC (34). Patients were selected based on predominance of PSMA-positivity on ^68^Ga-PSMA PET/CT, though no pre-defined SUV cutoff value was used. Of three eligible patients, two underwent 2 cycles of 6 GBq. One patient demonstrated partial response 5 weeks after the second treatment, with progression 7 months after. The other patient showed disease progression on ^18^F-FDG PET/CT 1 month after second treatment with stable disease at 6 months.

For hepatocellular carcinoma, two patients received ^177^Lu-PSMA-617 as part of a larger study evaluating the diagnostic capabilities of ^68^Ga-PSMA PET/CT (36). Unfortunately, dosimetric analysis showed accumulated doses 10-fold lower than typically achieved by 1 cycle of external beam radiation, and hence the therapy was stopped prior to a second cycle.

For breast cancer, one patient with triple-negative breast cancer refractory to other systemic agents received 2 cycles (7.5 GBq) of ^177^Lu-PSMA based on PSMA imaging (35). However, the patient progressed 4 weeks after cycle 2 and the treatment was stopped. Overall, these treatments were reported as well tolerated.

To address the broad range of cancers that express PSMA, there is new phase II basket trial NCT05867615 (LUBASKET) that recently began enrolling (**Table 2**). The trial does allow for inclusion of metastatic prostate cancer, so the overall non-prostate cancer enrollment potential is unclear.

## Discussion

With the recent FDA approval of ^177^Lu-PSMA-617 based on significant survival benefit in patients with mCRPC, there is significant interest in expanding PSMA-targeted RLT for other indications including non-prostate malignancies that express PSMA. Many of these cancers have much more limited treatment options than prostate cancer. For example, advanced salivary adenoid cystic carcinoma has no FDA-approved treatment options and has been shown to express PSMA in over 90% of patients in early studies (13). While most solid tumor express PSMA in neovasculature, there is also tumor cell expression of PSMA in ACC and other salivary malignancies, as well as some pancreatic cancers.

There are several potential concerns and limitations regarding RLT use for non-prostate cancers. A major issue continues to be heterogeneity of expression of PSMA, and the fact that the PSMA PET/CT SUV for non-prostate cancers tend to be markedly lower than for prostate adenocarcinoma (23). In prostate cancer, lower and heterogeneous PSMA expression on PSMA PET/CT imaging has been shown to correlate with lower response rate (61). A complicating factor here is the fact that unlike for prostate cancer, in non-prostate cancer there is not consistent correlation between expression of PSMA on IHC and signal on PSMA PET/CT (13). This suggests a lack of appreciation for all the factors that contribute to PSMA-ligand binding and accumulation in tumor tissue and neovasculature. Furthermore, response to PSMA RLTs in the limited non-prostatic cases discussed does not consistently associate with lesion SUV_max_ (14, 23, 25, 37, 59). There may be also different kinetics and distribution of binding for diagnostic ^68^Ga-PSMA ligands versus RLT ligands, which may be influenced by factors including tissue location, an important consideration for tumors like soft tissue sarcomas. Also problematic are dosimetric studies showing variable lesion uptake within and across studies (24, 25, 37), the underlying cause of which is unclear. It is promising that across tumor types there do appear to be cases in which PSMA RLT show good uptake on scintigraphy and partial metabolic responses (14, 23–25, 30). Dedicated dosimetry studies will continue to be essential for PSMA RLT in non-prostate cancers to determine absorbed dose to tumors and normal organs at different administered activities.

Another concern is optimal patient selection. As noted above, PSMA PET/CT imaging has potential for patient selection but needs to be further validated as a response biomarker for non-prostate malignancies. The European Association of Urology (EAU) and European Association of Nuclear Medicine (EANM) recently collaboratively released consensus guidelines regarding usage of PSMA PET/CT imaging for RLT applications (62). There was unanimous recommendation to use PSMA PET/CT imaging to demonstrate expression for patient selection, though no specific cutoffs were postulated. ^68^Ga-PSMA-11 and ^18^F-DCFPyL were the preferred PET tracers. The utility of ^18^F-FDG PET for patient selection has been demonstrated for prostate cancer but is an unanswered question for non-prostate cancers. A recent retrospective analysis by Seifert et al. found that in metastatic CRPC, mismatches in detection favoring ^18^F-FDG were rare (3%) compared to those favoring PSMA PET/CT (18%). However, studies specific to non-prostate cancers may be needed, as supported by the data from Wang et al. in ACC showing that detection mismatches favored ^18^F-FDG for lung metastases (52).

Specific PET criteria should be investigated for balancing likelihood of response while not excluding patients who may benefit. Data from prostate cancer RLT trials suggest that baseline PET uptake parameters such as mean and max SUV are associated with outcomes (63, 64). Currently for non-prostate cancers, patient selection tends to include patients with more advanced stage and comorbidities. This may limit completion of enough cycles to achieve therapeutic benefit, as seen in the pilot studies in non-prostate cancers to date. Therefore, prospective phase II-III trials with further inclusion and exclusion criteria in addition to dosimetric analysis are needed and may be more likely to show treatment response and yield information regarding PET screening parameters. There is interest in developing trials with criteria for target expression rather than specific disease indications (65, 66), and there is precedent for FDA approval of therapy based on biomarkers (e.g. pembrolizumab for microsatellite instability-high solid tumors) (67).

A third concern is limited data on dose-limiting toxicities. Thus far, ^177^Lu-PSMA-617 and ^177^Lu-PSMA-I&T have demonstrated favorable safety profiles for metastatic CRPC patients in large retrospective cohorts (68). Renal doses for both compounds were <1 Gy/GBq, with 1-1.5x higher accumulation for ^177^Lu-PSMA-I&T across studies, with no reports of grade 3 nephrotoxicity (68–70). While there has been concern about renal toxicity, a recent study of metastatic CRPC patients with at least CKD stage 3 showed that RLT (at least 2 cycles of ^177^Lu-PSMA-617; median 5 cycles) led to stable or improved GFR in most patients (71). However, RLT should still be administered with caution given reports of patients developing renal thrombotic microangiopathy following extensive treatment (72). Analysis of the VISION trial demonstrated that ^177^Lu-PSMA-617 treatments were associated with additional risk of grade 3 and 4 hematologic side effects, including anemia, lymphopenia, and thrombocytopenia (73). For ^177^Lu-PSMA-I&T, incidence of grade 3 or 4 toxicity is less than 5% (70). Different conjugates like ^177^Lu-EB-PSMA-617 may have better target accumulation, but also have increased uptake in normal organs like the normal salivary glands, kidneys, and bone marrow (74). Xerostomia occurs in approximately 30% of patients after ^177^Lu-EB-PSMA-617 ^(^ 75), which is concordant with numbers from ^177^Lu-PSMA-617 use in the VISION trial (73). Effects could be more severe in patients who have had prior salivary gland resection or external beam radiation. Receipt of prior chemotherapy such as taxanes can also predispose patients to more significant hematopoietic toxicities (76).

In general, salivary gland cancer (SGC) is likely to be the first non-prostate cancer investigated in a prospective randomized trial as a potential indication for PSMA RLT, and pilot studies in SGC have shown some of the challenges noted above. Early results of PSMA RLTs in SGC have shown a less profound impact of PSMA RLTs compared to prostate cancer. The potential reasons for this are multifactorial including potential differences in PSMA expression, radiosensitivity, and patient selection in the pilot studies. The mean ^68^Ga-PSMA SUV_max_ for SGCs noted above tend to be lower in comparison to mean SUV_max_ reported in prostate cancer (median of 13.3) (77). In a combined analysis of PSMA PET/CT studies in SGC, Tan et al. reported overall local recurrence and metastatic mean SUV_max_ of 6.33 [2.41-13.8] and 6.82 [2.04-14.9] respectively for ACC; those respective mean SUV_max_ values for other SGCs (largely SDC) were 10.57 [4.0-16.8] and 6.06 [1.43-14.27] respectively (20). Of the 29 patients with SGC who have received PSMA RLT in pilot studies, 11 received 3 or more cycles on protocol. This contrasts with recently reported prospective studies in prostate cancer, as patients received a median of 5 cycles (37.5 GBq) in both the TheraP and VISION trials. This may be related to the selection criteria and late stage of patients with SGC treated in the pilot studies, and prospective phase II studies may show more favorable response. Interestingly, two ACC patients with partial metabolic response were among those that completed 3 or more cycles, and both had intracranial metastases (14, 23).

Overall, there is significant promise for PSMA-targeted therapy in non-prostate malignancies. Even if there is lower absorbed tumor dose due to lower and more heterogeneous PSMA expression, there may be significant treatment response in comparison with currently available treatment options for these malignancies. Further study is indicated investigating PSMA RLT dosimetry, PSMA PET/CT imaging as a biomarker in non-prostate cancers, and prospective phase II and III trials will be required for assessment of PSMA RLT treatment safety and efficacy.

## Author contributions

Conceptualization, writing, editing, and final approval of manuscript: JW and AK.
