# Supplementary figures and images for: PSMA-targeted therapy for non-prostate cancers
Source: Front Oncol. 2023 Aug 14;13:1220586. doi: 10.3389/fonc.2023.1220586 (PMC10461313; doi:10.3389/fonc.2023.1220586)

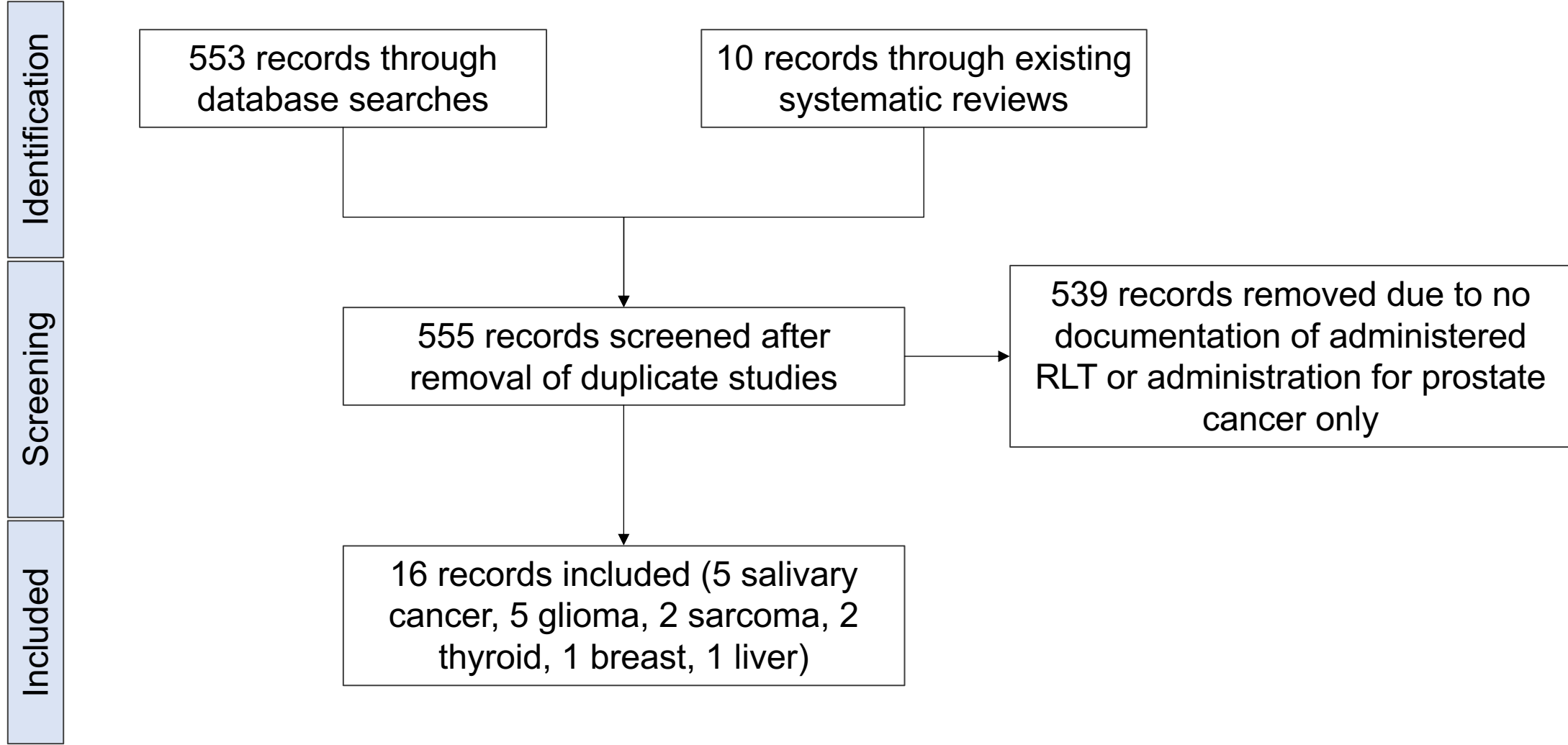

Supplemental Figure 1. PRISMA diagram.

Supplement: Supplementary file 1 [file DataSheet_1.pdf]
